# 加速康复外科——临床应用加减之间？

**DOI:** 10.3779/j.issn.1009-3419.2019.11.01

**Published:** 2019-11-20

**Authors:** 国卫 车

**Affiliations:** 610041 成都，四川大学华西医院胸外科 Department of Thoracic Surgery, West China Hospital, Sichuan University, Chengdu 610041, China

**Keywords:** 加速康复外科, 人文因素, 医疗服务, 医疗干预, Enhanced recovery after surgery, Humanistic factor, Medical service, Medical intervence

## Abstract

加速康复外科（enhanced recovery after surgery, ERAS）理念诞生与微创外科技术进步密切相关。ERAS发展过程中利用技术，又融入人文因素，使其高于技术，而丰富ERAS的内涵和外延。结合近年ERAS在外科各领域的进展，总结ERAS临床应用的现状与策略：一是微创技术进步使ERAS理念的临床应用成为必然；二是外科学理论与技术的发展，深入与扩大了ERAS的内涵与外延；三是ERAS临床应用需要我们更新观念；四是ERAS的临床实践需要在医疗服务和医疗干预之间合理选择；五是ERAS临床应用加减之间应用的现实问题与策略。从而深入理解ERAS的真正临床意义，更好地指导临床实践。

## Abstract (en)

The foundation of enhanced recovery after surgery (ERAS) is closely related to the minimally invasive surgery progress. In the development of ERAS, technology is utilized and humanistic factors are integrated to make it higher than technology, thus enriching the connotation and extension of ERAS. Based on the progress of ERAS in various fields of surgery in recent years, the current situation and strategies of clinical application of ERAS are summarized. First, the clinical practice of ERAS is inevitable for minimally invasive surgery progress. Second, the development of surgery theory and technology has deepened and expanded the connotation and extension of ERAS. Thirdly, the clinical application of ERAS requires us to renew our ideas. Fourth, the clinical practice of ERAS requires reasonable choice of medical service and medical intervention. The fifth is to the practical problems and strategies in clinical application of ERAS, to understand the complete clinical significance of ERAS, and better guide clinical practice.

21世纪微创技术发展促进外科学进步，集中体现在加速康复外科（enhanced recovery after surgery, ERAS）理念的临床应用和推广^[1]^。ERAS理念和微创外科技术相互影响，共同促进外科手术向更低风险和更小创伤发展。ERAS理念已深入到外科各个专科（普通外科、胸心外科和神经外科），已发表临床数据均显示应用ERAS方案可降低术后并发症，缩短住院时间，减少医疗费用^[2]^。但各个专科ERAS应用的广度和深度差异显著，如结直肠外科发展快，效果也好，同时制订了相应的临床共识与指南^[3]^。而其他专业（如胸心外科）相对发展较慢，原因可能与各专业有其自身的特殊性有关外，也可能与外科医生对ERAS理念的理解和临床应用的切入点选择有关^[4]^。本文结合最近几年ERAS发展及取得的临床效果，探讨ERAS理念如何更好地在临床上应用。

## 1 ERAS的临床应用是外科学发展的必然

现在看来，当时的理发师，以放血和切除病灶为主，理发外科仅仅是除疾^[5]^。消毒、麻醉、输血、抗感染和外科技术的发展，使外科治疗的领域越来越多，当需要或通过外科能够治愈更多的疾病时，尤其是肿瘤需要通过手术才能治愈时，外科学已远远超越手术的范畴，而是一门真正的科学，不但关注术中并发症，更加关注延长患者生命和改善患者生活质量^[5, 6]^。从理发外科到科学外科，经历了近千年的发展和完善^[5, 7]^。

微创技术应用于手术治疗既扩大和丰富了外科学理论，也促进了ERAS从理论走向临床实践。回顾胸外科尤其是肺外科的发展历程，20世纪80年代以前，囿于手术器械和药物的限制，主要是解决切除肿瘤、治疗疾病；1980年-2000年，伴随手术器械和药物的更新，不仅扩大了手术范围，也降低手术相关并发症，并通过外科手术提高了肿瘤患者的生存率，早期肿瘤效果更加显著^[8]^。21世纪以来，微创外科与ERAS理念相互融合、相互促进，不但缩短住院时间，降低围手术期的并发症，减少住院费用，也可改善患者的就医体验^[9]^。1992年，以胸腔镜肺叶切除术开展为契机，使ERAS的理念在肺外科得到快速进步，尤其是近10年，胸腔镜肺叶切除术的应用得到普及，并成为肺癌肺叶切除可选择的术式，也使肺术后的加速康复从各方面得到体现：肺部感染等并发症显著降低，住院费用明显减少，住院时间显著缩短等^[10]^。

ERAS无风险、无痛苦是外科学发展的最终目标。微创外科技术和理论是达成这个目标的有效手段。诊断技术（影像技术、血液检测技术）的发展，使许多无症状的肿瘤患者早期发现，而早期肿瘤患者治愈的唯一治疗手段是手术，手术创伤成为降低患者生活质量（躯体和心理的影响）的主要因素^[11, 12]^。当今外科应该在既考虑使患者长期生存，又不影响生活质量的前提下计划手术方式，而这正是ERAS理念的充分体现，因此，ERAS是外科学发展到今天的必然结果^[13]^。

## 2 ERAS内涵与外延需要加深与扩大

ERAS理念（no risk and no pain）目前更多关注的是围手术期临床治疗及效果，如术前宣教、饮食、微创手术方法和降低并发症等^[14]^；ERAS需要我们对患者的关注从围手术期扩大到全生命周期；从降低并发症深入到不影响术后生活质量，使患者回归正常生活与工作^[15, 16]^。基于ERAS理念的深入与扩大，临床上如何理解与操作呢？

无风险（no risk）主要是强调术前评估、预防和精准治疗。术前客观、正确评估患者的病史、病情、伴随疾病及治疗史，然后进行合理与精准的术前预防治疗，如戒烟、肺功能差需要肺康复训练，术前抗凝预防肺栓塞等^[17-19]^；病情评估选择精准治疗方式（单孔或多孔胸腔镜肺段、肺叶切除等）。多学科协作选择合理的临床诊断与预防措施，降低并发症的发生；精准的手术操作，避免不必要的手术创伤，如肺癌手术清扫第7组淋巴结时，尽量保留迷走神经主干和健肺迷走神经肺支，再如左肺下叶的肺微小结节，是否必要清扫第5、6组淋巴结等，这些均有可能影响患者长期生活质量，而不影响患者的生存^[20, 21]^。

无痛苦（no pain）包括躯体的疼痛或不适和心理创伤。现在躯体创伤来源于疾病本身（症状）越来越少，主要是医疗治疗措施导致，如麻醉过程（气管插管、监测仪器等）和手术本身（疼痛、头晕、恶心或呕吐、胃肠功能紊乱、气短或疲劳等）^[22, 23]^。心理创伤可能是多方面的，且目前可能比躯体创伤更重；心理创伤的影响是多方面的：一是患者自身对疾病的恐惧；二是对治疗过程的担心（如麻醉和手术过程，尤其是术前谈话后）；三是对治疗结果的不确定性（是否会有严重并发症或能否彻底治愈等）；四是医疗活动确实带来的术后相关并发症的恢复过程（如声嘶、顽固性疼痛等）^[24]^。

总之，ERAS需要根据自身的医疗条件（医护和医院平台），结合患者病情，选择最合理的治疗方案，以期围手术期达到避免或降低并发症，远期达到不影响患者生活质量的目标。

## 3 ERAS的发展需要不断更新观念

微创技术的进步、影像学和腔镜技术的进步、靶向和免疫治疗药物的出现。直接导致外科治疗方式的根本性变革，同时需要外科治疗的患者群体发生变化。表现在早期患者增多，晚期患者减少。通过外科治疗早期肿瘤患者，生存时间更长（或不影响寿命），影响患者术后生活质量的不是疾病自身，而是手术创伤。如何预防、降低或改善或因医疗活动治疗疾病导致患者生活质量降低，成为目前不得不思考的现实问题。这不但需要我们对ERAS理论的内涵和外延有更深的认识，也需要我们在临床工作中更新观念（表 1）。

**1:** 加速康复外科相对应的治疗新观念 The renew concept of enhanced recovery after surgery

	Concept (old)	Concept (new)	Measures
Object	Treatment of diseases	Recovery and quality of life	Precise assessment
Model	Single discipline	Multidisciplinary collaboration	Team building
Tactics	Post-event treatment	Prior prevention	Prior assessment
Method	Technology first	Technical and humanistic care	Reduce medical intervention
Process	Personal medical treatment	Humanized service	Increase medical services

微创外科技术和肿瘤的早期发现需要解决患者长期生存的同时，更加关注患者的生活质量（心理和躯体健康）^[25]^。外科手术治疗更加精准化，手术治疗目的由袪除疾病向使患者更快康复和提高生活质量转变；临床治疗模式由单一学科向多学科协作转变，充分发挥各专科的优势服务病人，如呼吸科或康复科术前评估与预防性治疗伴随疾病、营养科制订围手术期个性化餐食、麻醉科（麻醉方法、是否气管插管等）及手术室（合理器械准备及监测）个体化的准备、中医科和疼痛科术后疼痛及胃肠功能、咳嗽的及时处理等^[26-29]^。多学科的协作可达到事后性处理的策略真正向事前性预防转变，同时使各学科诊治端口前移，如术前具有血栓形成高危因素的患者，术前应用抗凝药，有效降低了围手术期肺栓塞的发生。结合当前临床实践模式，真正实现创伤最小化、效益最大化的目标，充分利用医疗技术的同时也要重视人文化的服务；既追求技术的先进，又注重患者自身感受；医疗过程中既有个体化的治疗手段，也有人性化服务，使治疗效果和生活质量同时改善。实现ERAS的目标需要我们在医疗活动中合理取舍医疗服务和医疗干预。

## 4 ERAS临床应用的“加减”法

ERAS不是简单的加减法，而是合理取舍。临床应用中具体指的是医疗服务做加法，改善患者就医体验；医疗干预做减法，降低风险与痛苦。医疗过程重点在围手术期，术前客观评估，精准准备与预防，体现客观和可操作性；术中强调优化：麻醉方法及操作流程、手术方案及技术流程、监测指标与器械准备，以观察指标客观精准、缩短手术时间和保证安全为目标；术后需要简化：按需调整监控指标、管道安放、液体输注和相关检查等，体现个体化和人性化相结合的服务流程。

术前评估：有许多有创检查，这些是否完全有必要？以肺癌为例，目前的检查都是基于晚期肺癌准备，如纤维支气管镜和全身骨扫描检查。对于直径 < 3 cm肺结节，术前检查是否都需要，值得思考？高龄患者，无明显心血管疾病，是否一定要做心导管冠脉造影，能否通过心肺运动试验进行？术前评估重视病史及可能影响医疗过程的相关疾病及治疗史，选择合理的检查手段，如肺小结节，低剂量螺旋计算机断层扫描（computed tomography, CT），分析小结节的位置、实性成分的比例、体积和重量，并利用计算机软件推算肿瘤的倍增时间，以助于制订合理的治疗方案。术前的宣教应该转向“主动参与”的个体化宣教，针对患者心中疑惑进行针对性的讲解，对患者需要配合的操作，进行讲解或示范（如呼吸训练器目标值的设定、术中不留置尿管时如何配合及注意事项等）^[30-32]^，以发现和预防问题导向，进行必要的有创性检查，增加医疗过程中可能出现问题的预防宣讲及如何处理为主（医疗服务）。

术中优化：优化麻醉方法和流程，如术前评估手术难度不大，时间不长及伴随疾病少，是否可以考虑不选用全麻气管插管（如非气管插管进行气胸、手汗症手术等），可否不选择有创血压监测、留置尿管等。若术中顺利，可否手术室或恢复室拔出气管插管，甚至在手术室拔出胸腔引流管等。术中操作器械，因超声刀和切割性缝合器的大量的应用，习惯性应用的手术器械是否需要常规准备，可否按需准备（模块化或个体化配置）^[27, 28]^。这些均可有效节约手术时间，也有助于减少创伤。

术后简化：按需或定时提供相应监测。术后早期下床活动，需要减少不必要的监控和管道安放。如术后心电监护（医疗干预），若不安装，需要护士每2 h进行床旁血压监测（医疗服务）等，无尿管留置，需要护士关注并采取措施使患者第一次小便顺利排出，胸腔引流管不留置或早期拔出都需要医护人员采用相应安全措施（如超声和影像检查等）^[34, 36, 37]^，这些均需要增加医护人员提供更多的服务，也增加了他们的工作量。

临床工作中的加（医疗服务）减（医疗干预）之间，充分体现加速康复外科内涵。提供个体化和人性化服务，并保证患者安全的前提下，无形之中加大了医护工作人员的工作量和强度，也使医护工作人员业务风险增加，因此更需要团队合作。

## 5 临床工作“加减”应用中的困境与策略

微创外科技术和ERAS理念相互促进，共同发展，使外科学的发展进入了一个新阶段^[36]^；实现加速康复外科目标，还有很长的路要走。目前在临床工作中充分实践ERAS的理念仍面临诸多问题：首先是现有的临床诊治规范与已形成的工作习惯同加速康复理念的冲突^[37]^；现有的各种规范、指南和共识的规定，已不完全适应于目前外科学发展，可新的指南或规范尚未出台，或缺乏循证医学证据。其次是ERAS需要团队建设和多学科协作，而多学科协作模式及运行过程仍不成熟。最后是ERAS实施过程中，尤其是增加的医疗服务，无法收费；目前的医疗收费项目，多是基于医疗干预过程中的材料费用，而忽略了医疗服务费用，也是推广慢的主要原因之一。如何解决现实与ERAS发展之间的矛盾呢？近20年的探索与实践，也形成了一些策略：一是全球各大医学中心，已进行了大量的临床研究，并得到了客观的循证医学证据，并初步发表了指南与共识（集中于普通外科，如结直肠、肝胆等），在其他外科分支如胸外科、心脏外科等，可在现有ERAS理论指导下，利用成熟的临床经验，进行大量的临床研究；二是根据各家医院的情况，组建适合的ERAS团队，如国内比较成熟的四川大学华西医院模式（以病人为中心，多学科协作模式），以浙江大学第一附属医院模式（ERAS病房），均取得了显著效果。三是减少医疗干预的同时，也极大的节省了医疗费用，而医疗服务费用没有增加，使ERAS多数停滞在科研阶段，难以推广。需要我们在临床科研工作中，合理分析减少医疗干预时的节约的费用，同时统计增加医疗服务的工作量。医院也可考虑以服务包的形式申请。

总之，ERAS是外科学发展的方向，其理论和目的是以病人为中心，整合医学理论发展的先进成果，需要我们不断地总结临床实践并服务于医疗工作中，必将推动ERAS目标no risk and pain的实现，同时极大地改善患者的就医体验，服务于健康中国。
